# Preclinical and Clinical Development of a YFV 17 D-Based Chimeric Vaccine against West Nile Virus

**DOI:** 10.3390/v5123048

**Published:** 2013-12-09

**Authors:** Gustavo H. Dayan, Konstantin Pugachev, Joan Bevilacqua, Jean Lang, Thomas P. Monath

**Affiliations:** 1 Sanofi Pasteur, 1 Discovery Drive, Swiftwater, PA 18370, USA; 2 Sanofi Pasteur, 38 Sidney St, Cambridge, MA 02139, USA; E-Mail: Konstantin.Pugachev@sanofipasteur.com; 3 Sanofi Pasteur, 1755 Steeles Ave West, Toronto, ON M2R 3T4, Canada; E-Mail: Joan.Bevilacqua@sanofipasteur.com; 4 Sanofi Pasteur, 1541 Avenue, Marcel Mérieux, Marcy-l'Étoile 69280, France; E-Mail: Jean.Lang@sanofipasteur.com; 5 Acambis Inc., 38 Sidney Street, Cambridge, MA 02139, USA

**Keywords:** chimeric vaccine, West Nile virus vaccine, yellow fever vector vaccine, pre-clinical, clinical development

## Abstract

Substantial success has been achieved in the development and implementation of West Nile (WN) vaccines for horses; however, no human WN vaccines are approved. This review focuses on the construction, pre-clinical and clinical characterization of ChimeriVax-WN02 for humans, a live chimeric vaccine composed of a yellow fever (YF) 17D virus in which the prM-E envelope protein genes are replaced with the corresponding genes of the WN NY99 virus. Pre-clinical studies demonstrated that ChimeriVax-WN02 was significantly less neurovirulent than YF 17D in mice and rhesus and cynomolgus monkeys. The vaccine elicited neutralizing antibody titers after inoculation in hamsters and monkeys and protected immunized animals from lethal challenge including intracerebral inoculation of high dose of WN NY99 virus. Safety, viremia and immunogenicity of ChimeriVax-WN02 were assessed in one phase I study and in two phase II clinical trials. No safety signals were detected in the three clinical trials with no remarkable differences in incidence of adverse events (AEs) between vaccine and placebo recipients. Viremia was transient and the mean viremia levels were low. The vaccine elicited strong and durable neutralizing antibody and cytotoxic T cell responses. WN epidemiology impedes a classical licensure pathway; therefore, innovative licensure strategies should be explored.

## 1. Introduction

West Nile (WN) virus is a human pathogen in the *Flavivirus* genus of the *Flaviviridae* family, which also includes Japanese encephalitis (JE), yellow fever (YF), dengue (DEN) and tick-borne encephalitis (TBE) viruses [1]. It is transmitted by mosquitoes, with wild birds being the main natural host. Based on antigenic cross-reactivity, the virus is grouped in the JE complex of flaviviruses together with other human pathogens including JE, St. Louis encephalitis (SLE), Rocio (ROC), and Murray Valley encephalitis (MVE). The human disease caused by WN virus varies from dengue-like illness to fatal meningoencephalitis, with the elderly most likely to have severe illness. Since the introduction of WN virus in 1999 to the New York City area, the virus has rapidly spread through North America, the Caribbean and Mexico, and has reached continental South America. It was initially concluded that the strain imported into the US originated in the Middle East [2], which however was questioned more recently in that it is possible that both the NY99 strain and its Middle Eastern suspected parent may have originated at an earlier time point from the same, likely African, ancestor [3]. In the US, disease incidence peaked in 2003, with 9,862 reported cases, approximately one-third of which were accompanied by neurological symptoms, and 264 deaths. Following a decline, the incidence was again on the rise in 2012, with at least 5,674 cases and 286 deaths [4]. This illustrates the cyclical nature of epidemics of mosquito-borne encephalitis in the USA, and the continuing need for effective public health interventions.

WN virions are spherical particles of approximately 50 nm in diameter. The genome is a single-stranded RNA molecule of positive polarity, about 11,000 nucleotides (nt) in length. It contains a single long open reading frame (ORF) flanked by 5' and 3' untranslated terminal regions (UTRs). The ORF encodes a polyprotein precursor C-prM/M-E-NS1-NS2A/2B-NS3-NS4A/4B-NS5 that is cleaved co- and post-translationally into individual viral proteins, the structural proteins C (capsid), prM/M (pre-membrane/membrane) and E (envelope), and several non-structural (NS) proteins essential for virus replication. The E protein is the main functional protein of the envelope responsible for virus binding to cellular receptors and membrane fusion. It is also the main antigen, eliciting neutralizing antibodies that are considered to be the main correlate of protective immunity [5]. Cellular immunity is also an essential component of adaptive immunity. Virus-specific CD8^+^ and CD4^+^ T-cell epitopes occur throughout both the structural and NS proteins, although they mostly concentrate within E and NS3. 

There are no antiviral drugs for the treatment of WN disease. A variety of compounds show promise *in vitro* [6], but no clinical data are available. Some evidence suggests that passive administration of intravenous globulin containing high titer WN antibodies may have therapeutic activity in animal models [7]; however, despite some case reports to the contrary, no clear benefit from passive immunotherapy was evident in humans when compared to placebo [8]. Vector control measures are mostly used to prevent outbreaks. However, outbreaks still occur and vector control is often not possible or practical in low-population density areas that experience high WN virus incidence. Therefore, vaccination of people at risk could be the most effective means of protection against WN virus disease. Licensed vaccines that are currently available for use in humans against flaviviruses include JE, TBE, and YF and have been extensively reviewed [9,10,11]; however, no approved human vaccine is available against WN. The emergence of WN in North America has spurred extensive interest in the development of human and veterinary vaccines. Several human vaccine candidates have been investigated (Table 1). 

**Table 1:** West Nile (WN) vaccines for protection of humans, by the company or institute developing the vaccine and the stage of development.

Company/Institute (Originator)	Vaccine type	Vaccine	Stage of development
Sanofi Pasteur (Acambis)	Live, attenuated	Chimeric YF vector, WN prME	Phase II
National Institutes of Health (USA)	Live, attenuated	Chimeric Den 4 vector, WN prME	Phase I
Vical	DNA	Plasmid DNA encoding WN prME proteins, no adjuvant	Phase I
Takeda (Inviragen)	Live, attenuated	Chimeric Den2 vector, WN prME	Preclinical
Institut Pasteur	Live, attenuated	Measles vector, WN E	Preclinical
Institut Pasteur	Live, attenuated	Lentivirus vector, WN E	Preclinical
Johnson & Johnson (Crucell)	Inactivated virus	Formalin inactivated whole virion	Preclinical
Intercell	Inactivated virus	Formalin inactivated whole virion	Preclinical
Baxter Biosciences	Inactivated virus	Formalin inactivated whole virion	Preclinical
Kanonji Institute (Osaka University)	Inactivated virus	Formalin inactivated whole virion	Preclinical
Hawaii Biotech	Subunit	WN E protein expressed in Drosphila cells	Preclinical
L2 Diagnostics	Subunit	WN E protein expressed in Sf9 cells	Preclinical
University of Texas Medical Branch	Replicon	Single cycle WN, capsid deleted	Preclinical
National Institutes of Health (USA)	Virus like particles	WN CprME coexpressed in baculovirus	Preclinical

Substantial success has been achieved in the development and implementation of WN vaccines for horses, which previously suffered WN disease with an incidence nearly 70 times higher than humans (Table 2).

One of the licensed vaccines, ChimeriVax-WN vaccine for horses (PreveNile®, Intervet, Dover, DE, USA), is a live chimeric vaccine where the live attenuated YF 17D vaccine virus is used as a vector in which the prM-E and envelope protein genes are replaced with those from the WN virus, and is the only single dose vaccine available against West Nile [12,13]. The live vaccine was taken off the market temporarily because of allergic reactions to an excipient (stabilizer) in the vaccine; these reactions were not due to the vaccine virus itself, and the allergenic material has now been removed. The human vaccine candidate described below does not contain the allergenic excipient. A similar vaccine is under development for humans, representing an integration of human and animal health objectives. This review will focus on the construction and pre-clinical and clinical characterization of a live chimeric vaccine, ChimeriVax-WN for humans. 

**Table 2:** West Nile vaccines for horses approved in the United States.

Company	Vaccine	Brand name	Primary immunization
Intervet (Merck)	Live, attenuated, YF vector, WN prME (wild-type sequence)	PreveNile®	1 dose
Merial (Sanofi)	Live canarypox vector, WN prME transgene, adjuvanted	Recombitek® equine WNV vaccine	2 doses
Ft. Dodge Zoetis Inc. (formerly Pfizer Animal Health.)	Formalin-inactivated whole virus, adjuvanted	West Nile-Innovator®	2 doses
	Plasmid DNA	West Nile-Innovator DNA®	2 doses
Boeringer-Ingelheim	Formalin-inactivated whole virus	West Nile-Vetera®)	2 doses
Intervet (Merck)	Formalin inactivated YF/WN chimera, adjuvanted	EquiNile®	2 doses

## 2. Chimerivax-WN Construction

**Figure 1:**
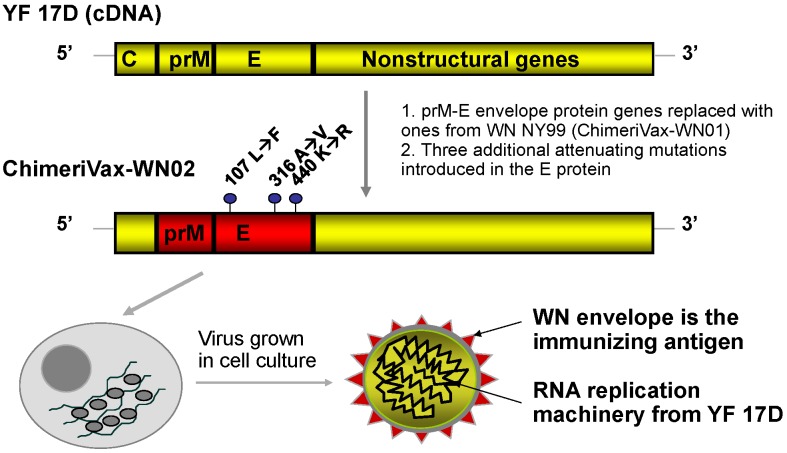
Construction of ChimeriVax-WN02.

The construction of live, chimeric vaccines against flaviviruses was an outgrowth of the cDNA technology [14] that permitted the switching of genes between different flaviviruses. The first such constructs were reported by Bray and Lai in 1991, who prepared intertypic chimeras of dengue viruses [15] and the first viable chimera between two genetically distant flaviviruses, DEN4 and TBE viruses, was reported by Pletnev *et al.* [16]. The ChimeriVax technology for creation of live vaccines against flavivirus diseases takes advantage of the live attenuated YF 17D vaccine virus as a vector in which the prM-E envelope protein genes are replaced with those from corresponding heterologous flaviviruses, resulting in highly attenuated and immunogenic chimeric viruses of the heterologous antigenic specificity (Figure 1) [17,18].

The YF 17D vaccine developed in the 1930s has been regarded as one of the most successful human vaccines, with more than 500 million doses administered worldwide [11]. Use of an existing vaccine as a vector for foreign genes provided a significant advantage, not only because of the expected high safety and immunogenicity of chimeric vaccines but also because it allowed the phenotypic characteristics of chimeras to be benchmarked against the parental virus.

YF 17D infects and activates antigen presenting cells, and induces robust and durable, possibly life-long immunity against YF after a single dose [11]. The ChimeriVax technology has been used in the construction of the ChimeriVax-JE (IMOJEV™, Sanofi Pasteur, France) vaccine for humans against JE, which is now a licensed product in use in Thailand and Australia (and is being licensed in several South-East Asian countries), the ChimeriVax-DEN vaccine which is currently in Phase III of development and ChimeriVax-WN. A similar vaccine was constructed against SLE virus [19]. The donor prM-E sequence of the WN vaccine candidate was derived from a strain isolated from the brain of a flamingo (*Phoenicopterus chilensis*) with fatal encephalitis at the Bronx Zoo, New York in 1999. The first vaccine candidate chimera, referred to as ChimeriVax-WN01, contained the wild-type WN NY99 prM-E sequence (Figure 1). This chimera was found to be significantly attenuated for mice when compared to both its WN NY99 parent and YF 17D. It was not neuroinvasive, but retained a degree of residual neurovirulence, causing approximately 25% fatal encephalitis in weanling mice after intracerebral (IC) inoculation [20]. ChimeriVax-WN01 was subsequently developed by Intervet as a vaccine for use in horses, which by virtue of host-range restriction are less susceptible to infection than primate species and therefore required a more active infection (higher level of *in vivo* replication and antigen expression) to elicit immune responses. To obtain a more attenuated vaccine candidate for human use, three attenuating amino acid changes were introduced into the E protein of ChimeriVax-WN01 at residues 107,316 and 440 [20]. The selection of these mutations was achieved by comparing the WN E gene sequence with that of the highly conserved closely related JE virus. The molecular basis for attenuation of an attenuated vaccine strain of JE (JE SA14-14-2) had been previously discussed [21], and the high homology of WN and JE allowed the identification of amino acid determinants in WN predicted to attenuate neurovirulence [12,22]. The chimeric virus with the mutated E gene was designated ChimeriVax-WN02 (Figure 1).

During development of the ChimeriVax-WN02 vaccine, a subpopulation of virus with a small plaque phenotype (SP) was identified in the vaccine lot produced under serum free condition in Vero cells at passage 5 (P5), the passage level used in the initial Phase I clinical study. The single mutation at M66 (LP) responsible for the SP phenotypic change appeared to be an adaptation to propagation of virus in serum free Vero cells. The two variants, the original large plaque (L➔P) and the SP viruses, were demonstrated to be highly attenuated with respect to mouse neurovirulence [20]. Because the SP virus generated lower viremia than the LP in a hamster model of WN infection but remained highly immunogenic in monkeys (see below), it was selected for production of vaccine lots to be used in Phase II clinical trials [18].

It should be noted that serious AEs associated with YF 17D vaccination, albeit very rare, have come to light with improved surveillance, such as YF vaccine associated neurotropic disease (YEL-AND) in adults (reporting rate ~0.8 per 100,000) and viscerotropic disease (YEL-AVD) resembling classical YF (reporting rate ~0.4 per 100,000, which could be higher in the elderly) [11]. Given that ChimeriVax-WN02 is built on a 17D backbone, these adverse events have been carefully followed. Although the number of subjects vaccinated with ChimeriVax-WN02 in clinical trials is insufficient to detect rare severe adverse events, no safety concerns were detected. It is also reassuring that no safety signals were detected with similar chimeric vaccines against Japanese encephalitis or dengue during clinical development that involves a substantially higher number of subjects [18]. The lower virulence of ChimeriVax-WN02 compared to YF 17D observed in animals also supports these results. 

## 3. Pre-Clinical Characterization

The biological properties of ChimeriVax -WN01 and -WN02 in animal models are summarized in Table 3 in comparison to the YF 17D vaccine. ChimeriVax-WN02 was significantly less neurovirulent than YF 17D in adult and suckling mice [19]. In particular, the suckling mouse neurovirulence test has been shown to represent a highly discriminating model for assessment of neurovirulence, and predictive of results in non-human primates [23].

Neuropathologic scores after IC inoculation of both rhesus and cynomolgus monkeys were significantly lower for ChimeriVax-WN02 than YF 17D virus (Table 3). There were no abnormalities in hematology and clinical chemistry, and no histological changes were observed in any examined peripheral organ of cynomolgus monkeys following IC or subcutaneous (SC) inoculation. For the uncloned P5 ChimeriVax-WN02, viremia was lower compared with YF 17D in rhesus monkeys, but higher in cynomolgus monkeys, yet within the WHO specifications established for YF 17D vaccine [20,24]. The latter observation was associated with a more pronounced early replication of ChimeriVax-WN02 in the skin inoculation site and lymph nodes. Generally, the biodistribution in monkeys of both ChimeriVax-WN02 and YF 17D viruses was similar, as demonstrated using sensitive quantitative PCR. Prominent sites of replication were skin and lymph tissues (as well as the spleen for YF 17D), generally sparing vital organs including the brain [24]. The chimera was highly immunogenic and protected immunized monkeys from lethal IC challenge with a high dose [5 log_10_ plaque-forming units (PFU)] of WN NY99 virus [20]. 

Hamsters are used as a model of WN virus infection. When given a single intramuscular (IM) inoculation of 3 or 6 log_10_ PFU of uncloned P5 ChimeriVax-WN02, hamsters developed significantly higher hemagglutination inhibition, complement fixing and neutralizing antibodies than hamsters receiving 2 doses of a commercial inactivated veterinary vaccine. Furthermore, hamsters inoculated with ChimeriVax-WN02 had sterile immunity when challenged with virulent WN whereas those given inactivated vaccine had break-through viremias and a rise in antibody titers after challenge [25].

The plaque-purified SP vaccine was tested in the hamster model, which revealed it to be more attenuated than the uncloned P5 ChimeriVax-WN02 virus, and evoked only low neutralizing antibody levels (Table 3). The hamster did not reliably predict immunogenicity in non-human primates or humans, however. Two studies were then performed using the SP variant in cynomolgus monkeys inoculated by the SC route. The SP virus was attenuated, as seen in hamsters, with an approximate 10-fold reduction in peak viremia titers compared to the uncloned P5 ChimeriVax-WN02 vaccine, and similar to viremia following YF 17D. Nevertheles,s neutralizing antibody titers elicited by the SP variant were robust and similar to those induced by the original uncloned vaccine (Table 3, Studies 2 and 3 in cynomolgus monkeys). In addition, a Good Laboratory Practice (GLP) study was performed in cynomolgus monkeys to determine the neurovirulence of the SP virus compared to YF 17D. As for the original uncloned P5 ChimeriVax-WN02 vaccine, the plaque purified SP virus was shown to be significantly less neurovirulent than YF 17D vaccine based on brain pathology observations (Table 3).

## 4. Clinical Development

Safety, viremia and immunogenicity of ChimeriVax-WN02 were assessed in one phase I study and in two phase II clinical trials [24,26,27]. Phase I evaluated a liquid frozen formulation of the uncloned vaccine produced at passage 5 in Vero cells, and phase II evaluated a lyophilized presentation of the plaque purified SP vaccine. Viremia was detected by plaque assay on Vero cell monolayers and antibody titers were measured in all studies using a 50% plaque reduction neutralization test (PRNT_50_) assay. 

### 4.1. Phase I Clinical Trial

This was a randomized double-blind, placebo controlled study where healthy adults aged 18–40 years were vaccinated with 5.0 log_10_ plaque-forming units (PFU) (n = 30) or 3.0 log_10_ PFU (n = 15) ChimeriVax-WN02, YF 17D vaccine (YF-Vax® (n = 5), or saline placebo (n = 30) by the SC route [24].

#### 4.1.1. Safety

In this study, ChimeriVax-WN02 was well tolerated with no observed differences in incidence of adverse events (AEs) between vaccine and placebo recipients. The percentage of subjects reporting at least one adverse event ranged from 83% in the placebo group to 100% in the YF vaccine recipients, being 87% and 93% among subjects receiving 5.0 log_10_ PFU and 3.0 log_10_ PFU ChimeriVax-WN02, respectively. No serious AEs related to vaccination were reported.

#### 4.1.2. Viremia

Viremia measured by the crystal violet plaque technique, was detected in 80% of YF vaccine recipients, 90% of 5.0 log_10_ PFU and 100% of 3.0 log_10_ PFU ChimeriVax-WN02 recipients (Table 4). Mean daily viremia levels were low (~100 PFU) for all 3 groups receiving vaccine; however, the peak viremia and the mean area under the curve (AUC) were statistically higher in subjects receiving the low dose. Similar observations have been made in the case of other ChimeriVax vaccines and are probably due to a lower innate and delayed adaptive immune response to the lower dose [28]. Viremia was highest 3–5 days after vaccination and cleared by day 10. No relationship between the level of viremia and the occurrence or severity of adverse events was found.

#### 4.1.3. Immunogenicity

At day 28 after vaccination, all but one subject (96%) in the 5.0 log_10_ PFU group and all subjects in the 3.0 log_10_ PFU seroconverted (PRNT titer ≥20). Geometric mean titers (GMTs) on day 28 after a single SC inoculation were high, and similar in both dose groups (1,218 in the 3.0 log_10_ PFU group and 1,280 in the 5.0 log_10_ PFU group) (Table 5). One year after vaccination, 100% of vaccinees in both groups were seropositive and titers had declined only about twofold to 595 and 640 in the 5.0 log_10_ PFU and 3.0 log_10_ PFU groups respectively. No correlation between neutralizing antibody response and viremia measured by the mean AUC was observed.

**Table 3:** Summary of preclinical characteristics of ChimeriVax-WN variants compared to YF 17D.

Model	Parameter	ChimeriVax-WN01 (veterinary vaccine)	ChimeriVax-WN02, Uncloned P5 vaccine virus	ChimeriVax-WN02, Cloned small plaque (SP) vaccine virus	YF 17D
Mouse (CD-1)	Neuroinvasiveness in adult mice after IP inoculation (at doses specified)	Mortality 0% (0.9–6.5 log_10_ PFU) ^[20]^			0% mortality (2.8–4.8 log_10_ PFU) ^[20]^
	Neurovirulence in adult mice after IC inoculation (at doses specified)	Mortality 25% (2.2–5.5 log_10_ PFU) ^[20]^	Mortality 11% (3.6 log_10_ PFU) ^a^		Mortality 100% (1–3.3 log_10_ PFU) ^a^
	Neurovirulence in 8 day old suckling mice after IC inoculation at doses 1.3–3.3 log_10_ PFU		Mortality 23% ^[20,23]^	Mortality 13% ^[20,23]^	Mortality 100% ^[23]^
	Neurovirulence in 6 day old suckling mice after IC inoculation at doses 1.3–3.3 log_10_ PFU			Mortality 50% ^b^	Mortality 100% ^c,b^
	Immunogenicity in adult mice: geometric mean (GMT) PRNT_50_ titers 28 days after SC inoculation at doses 3–5 log_10_ PFU	GMT 197 ^[20]^	GMT 20–37 ^[20]^		
	Survival (%) after wild-type WN99 challenge (3 log_10_ PFU IP) *vs.* 0% survival of mock immunized animals	Survival 100% ^[20]^	Survival 40% (for 3 log_10_ PFU vaccine dose) to 100% (for 5 log_10_ PFU vaccine dose) ^[20]^		
Hamster	Viremia after SC inoculation at 4–5 log_10_ PFU doses: % viremic, mean peak titers, duration		53% viremic, 350 PFU/mL, 1.2 days ^d,b^	20% viremic, 13 PFU/mL, 0.26 days ^b^	50% viremic, 33 PFU/mL, 0.5 days ^b^
	Immunogenicity after SC inoculation at doses 4–5 log_10_ PFU: % seroconversion, PRNT_50_ titers (GMT)		89% seroconversion, GMT 1016 ^b^	60% seroconversion, GMT 48 ^b^	100% seroconversion, GMT 15,521 ^b^
	Immunogenicity after IM inoculation of 3 or 6 log_10_ PFU: % seroconversion, PRNT_50_ titers (GMT)		100% seroconversion, GMT 299 ^[25]^		
	Viremia and survival (%) after WN99 challenge (4 log_10_ IP) of the above groups *vs*. 100% viremic and 50% survival for mock animals		10% viremic, 100% survival ^[25]^		
Monkey	Viremia after IC inoculation of rhesus or cynomolgus monkeys at ~5 log_10_ PFU dose (in neurovirulence tests): % viremic, mean peak titer, duration	**Rhesus ^[20]^**			**Rhesus ^[20]^**
		100%, 1.9 log_10_ PFU/mL, 4.5 days			100%, 2.65 log_10_ PFU/mL, 4.5 days
			**Cynomolgus**	**Cynomolgus**	**Cynomolgus**
			**Study 1:** 91%, 2097 PFU/mL, 2.9 days ^[20]^^,a^		**Study 1**: 91%, 357 PFU/mL, 2.5 days ^[20]^^,a^
				**Study 2:** 91%, 129 PFU/mL, 3.8 days ^b^	**Study 2:** 82%, 54 PFU/mL, 1.6 days ^b^
	Neurovirulence tests: combined brain pathology score	**Rhesus**			**Rhesus**
		0.49			0.6
			**Cynomolgus**	**Cynomolgus**	**Cynomolgus**
			**Study 1:** 0.13		**Study 1**: 0.52 (*p* = 0.0001) ^e^
				**Study 2:** 0.162	**Study 2:** 0.455 (*p* = 0.005)^e^
	Viremia after SC inoculation at 3–6 log_10_ PFU doses: % viremic (shown for cynomolgus monkeys), mean peak titer, duration		**Rhesus**		**Rhesus**
			1.4 log_10_ PFU/mL, 4.5 days ^[20]^		2.4 log_10_ PFU/mL, 3.5 days ^[20]^
			**Cynomolgus**	**Cynomolgus**	**Cynomolgus**
			**Study 1 ^f,^^[24]^:** 93% viremic, 474 PFU/mL, 3.7 days		**Study 1^f,^^[25]^**: 47% viremic, 67 PFU/mL, 1.4 days
			**Study 2 ^f,b^**: 100% viremic, 1925 PFU/mL, 5 days	**Study 2 ^f,b^**: 100% viremic, 18–90 PFU/mL, 1.5–5.7 days	
			**Study 3 ^f,b^:** 100% viremic, 1320 PFU/mL, 4.2 days	**Study 3 ^f,b^:** 100% viremic, 102–213 ^[5]^ PFU/mL, 3.7–6 days	
	Immunogenicity after SC inoculation at 3–6 log_10_ PFU doses: % seroconversion (shown for cynomolgus monkeys), PRNT_50_ (GMT) or mean log neutralization index (LNI) at indicated time points		**Rhesus**		**Rhesus**
			GMT 381 on day 30 ^[20]^		GMT >640 on day 30 ^[20]^
			**Cynomolgus**	**Cynomolgus**	**Cynomolgus**
			**Study 1 ^f,^^[24]^:** 100% seroconversion on day 14, GMT 2941		**Study 1 ^f,^^[24]^**: 90% seroconversion on day 14, LNI 1.97
			**Study 2 ^f,b^**: 100% seroconversion, GMT 3620 on day 31	**Study 2 ^f,b^**: 100% seroconversion, GMT 3620-4064 ^g^ on day 31	
			**Study 3 ^f,b^:** 100% seroconversion, GMT 32,510 on day 22	**Study 3 ^f,b^:** 100% seroconversion, GMT 11,494–14,482 ^e^ on day 22	
	Protection from wild-type WN IC challenge (5.4 log_10_ PFU) *vs.* unvaccinated controls 100% dead		**Rhesus ^[20]^**		**Rhesus ^[20]^**
			No viremia, no illness		100% viremic, 50% ill, 50% dead

^a^ Acambis IND BB-IND#11241, 2003; ^b^ Acambis IND BB-IND#11241, Amendment 2005; ^c^ Survival times were significantly longer for mice inoculated with ChimeriVax-WN02 than YF-VAX, *p* < 0.05 for all dose groups, log-rank test; ^d^ % viremic, mean peak viremia, mean duration viremia; ^e^ Comparing ChimeriVax-WN and YF-VAX, Kruskal- Wallis test; ^f^ Study 1: GLP tox study P5 vaccine; Study 2: SP P12 Production Seed Virus, pilot study; Study 3: P13 Vaccine lot, GLP tox study; ^g^ Range for different dose groups.

**Table 4:** Viremia in human subjects following vaccination with one dose of ChimeriVax-WN02.

	Mean Cmax (PFU/mL)	Mean AUC (PFU/mL)	Mean Duration (Days)	Percentage of viremic subjects * (%)
Phase I				
3.0 log_10_ PFU^a^ (n = 15)	187 (SD 165)	312 (SD 259)	4.7	100
5.0 log_10_ PFU^a^ (n = 30)	97 (SD 159)	173 (SD 252)	5.1	90
Phase II				
*WN003*				
*Part 1*				
3.0 log_10_ PFU^a^ (n = 24)	47 (95% CI 29, 77)	156** (95% CI 118, 206)	4.8	92
4.0 log_10_ PFU^a^ (n = 40)	33 (95% CI 23, 46)	138** (95% CI 113, 168)	4.1	90
5.0 log_10_ PFU^a^ (n = 31)	30 (95% CI 19, 48)	131** (95% CI 99, 173)	3.9	94
*Part 2*				
5.0 log_10_ PFU				
1–64 years ^b^ (n = 33)	25 (95% CI 17, 38)	115** (95% CI 94, 141)	3.7	85
≥65 years ^b^ (n = 31)	44 (95% CI 27, 72)	181** (95% CI 131, 249)	5.5	87
*WN004*				
3.0 log_10_ PFU ^b^ (n = 80)	43(95% CI 36, 53)	251(95% CI 219, 295)	5.9 ***	73
50–64 years	41(95% CI 31, 54)	240(95% CI 195, 295)	4.3 ***	57
≥65 years	46(95% CI 35, 60)	269.2(95% CI 219, 339)	7 ***	94
4.0 log_10_ PFU ^b^ (n = 82)	55 (95% CI 43, 69)	288 (95% CI 240, 347)	5.2 ***	74
50–64 years	53 (95% CI 37, 72)	275 (95% CI 219, 347)	4.4 ***	65
≥65 years	58 (95% CI 39, 85)	309 (95% CI 229, 417)	6.4 ***	93
5.0 log_10_ PFU ^b^ (n = 73)	51 (95% CI 41, 65)	269 (95% CI 234, 309)	4 ***	75
50–64 years	41 (95% CI 30, 56)	234 (95% CI 191, 288)	5.8 ***	72
≥65 years	65(95% CI 47, 87)	309(95% 257, 372)	3.2 ***	79

^a^ Measured by plaque assay with the crystal violet technique; ^b^ Measured by plaque assay with immunostain using a WN virus envelope protein specific monoclonal antibody; * Percentage of subjects with viremia; ** Day 1–14 after vaccination; *** Mean duration among subjects with quantified viremia (≥60 PFU/mL) 95% CI: 95% Confidence interval (lower bound, upper bound).

T-cell proliferative responses specific for WN were observed in peripheral blood mononuclear cells (PBMC) in 83% of 5.0 log_10_ PFU recipients and 87% of 3.0 log_10_ PFU recipients. The maximal stimulation index was measured on day 14 in 32% and 31% and on day 28 in 68% and in 69% of subjects vaccinated with ChimeriVax-WN02 5.0 PFU and 3.0 log_10_ PFU, respectively, who had detectable T lymphocyte lymphoproliferation [24]. Further analyses of the T cell responses in the Phase I trial were reported by Smith *et al*. [29]. CD8^+^ responses with a cytotoxic, polyfunctional effector cell phenotype were demonstrated. WN-specific CD8^+^ responses were found for the duration of the study, up to 1 year after vaccination. Over time, CD8^+^ cells evolved from effector function to a long-live memory phenotype.

In summary, the Phase I trial demonstrated that ChimeriVax-WN02 was well tolerated, caused low and transient viremia, and elicited strong and durable neutralizing antibody and cytotoxic T cell responses. The biological attributes seen in preclinical studies in hamsters and monkeys were confirmed in humans. The trial warranted further clinical evaluation, particularly in elderly subjects at highest risk of WN virus neuroinvasive disease. 

**Table 5:** Neutralizing antibody responses in human subjects 28 days following vaccination with one dose of ChimeriVax-WN02.

	n	Percentage of seroconversion (%)	PRNT_50_ * GMT
Phase I			
3.0 log_10_ PFU	14	100	1218 (SD 10,671)
5.0 log_10_ PFU	28	96	1280 (SD 7,895)
Phase II			
*WN003*			
*Part 1*			
3.0 log_10_ PFU	21	100	1367 (95% CI 711, 2629)
4.0 log_10_ PFU	37	97	2331 (95% CI 1193, 4554)
5.0 log_10_ PFU	28	96	3309 (95% CI 1727, 6342)
*Part 2*			
5.0 log_10_ PFU			
41-64 years	28	96	883 (95% CI 362, 2153)
≥ 65 years	27	96	965 (95% CI 442, 2106)
*WN004*			
3.0 log_10_ PFU	114	92	688 (95% CI 453, 1047)
50-64 years	69	90	585 (95% CI 331, 1033)
≥ 65 years	45	96	884 (95% CI 475, 1648)
4.0 log_10_ PFU	118	93	600 (95% CI 405, 890)
50-64 years	71	93	564 (95% CI 341, 932)
≥ 65 years	47	94	659 (95% CI 342, 1270)
5.0 log_10_ PFU	108	95	674 (95% CI 464, 978)
50-64 years	59	95	576 (95% CI 347, 955)
≥ 65 years	49	96	814 (95% CI 462, 1433)

* PRNT_50_: 50 % Plaque reduction neutralization test; 95% CI: 95% Confidence interval (lower bound, upper bound).

### 4.2. Phase II Clinical Trials

#### 4.2.1. WN003 Study

This was a randomized, double-blind, placebo-controlled, multi-center study conducted among healthy adults in the US. The study was done in two parts: Part 1 included adults aged 18–40 years who were vaccinated with ChimeriVax-WN02 3.7 > 10^5^ PFU (5.0 log_10_) (n = 31), ChimeriVax-WN02 3.7 × 10^4^ PFU (4.0 log_10_) (n = 40), ChimeriVax-WN02 3.7 × 10^3^ PFU (3.0 log_10_) (n = 24), or placebo (n = 17) [25]. The 5.0 log_10_ PFU dose was selected for Part 2 based on analysis of the immunogenicity, viremia, and safety data from Part 1. Part 2, comprised two age range cohorts, 41–64 years and ≥65 years; subjects in each age group were randomized to receive a single dose of 5.0 log_10_ PFU ChimeriVax-WN02 (n = 64, n = 33 41–64 years and n = 31 ≥65 years) or placebo (n = 32, n = 15 41–64 years and n = 17 ≥65 years). 

##### 4.2.1.1. Safety

The safety and tolerability of this vaccine were also evidenced in this study. In Part 1, the percentage of subjects with AEs and adverse reactions (ARs) (*i.e.*, treatment-related AEs) was highest in the placebo group (AEs: 82% and ARs: 53%) and were similar across the vaccine groups (AEs: range 67%–71%, and ARs: range 23%–33%). Most common AEs reported 28 days after vaccination were headache and fatigue. The favorable safety profile among the elderly was demonstrated for the first time in this study. In fact in Part 2, fewer subjects experienced AEs or ARs in the ≥65 years cohort (AEs: 74% and ARs: 52%) compared to the 41–64 years cohort (AEs: 94% and ARs: 82%). Most AEs were mild or moderate in severity and all serious AEs in the study were considered unrelated to study vaccine.

##### 4.2.1.2. Viremia

In Part 1, most subjects in the study (~90%) experienced a transient low viremia measured by plaque assay with the crystal violet technique. Mean daily viremia levels were low for all 3 groups receiving vaccine; however, the peak viremia and the mean AUC tended to be higher in subjects receiving the low dose, as noted in Phase I (Table 4). Viremia levels peaked around Days 6 and 7 for the 5.0 log_10_ PFU group and on Day 5 for the other two vaccine dosage groups. The mean duration of viremia ranged from 3.9 days in the 5.0 log_10_ PFU group to 4.8 days in the 3.0 log_10_ PFU group. In Part 2, viremia measured by plaque assay, using an immunostain with a WN virus envelope protein specific monoclonal antibody, was detected in 87% of subjects ≥65 years and in 85% subjects 41 to 64 years (Table 4). Mean daily viremia levels were low in both age groups and somewhat higher in ≥65 years old subjects. Peak viremia and mean AUC also tended to be higher in the ≥65 years cohort. Viremia peaked around days 6 and 7 after vaccination and the mean duration tended to be a longer in the ≥65 years cohort (5.5 days) compared to the 41–64 years cohort (3.7 days). A higher proportion of viremic elderly subjects and more prolonged viremia compared to young participants was also observed in a prior study with the YF-17D vaccine [30]. 

There were no clear correlations between days of viremia and the occurrence of systemic reactions during the study.

##### 4.2.1.3. Immunogenicity

After a single inoculation, almost all subjects developed high titers of WN-specific neutralizing antibody titers by day 28, as in the prior study [24]. In Part 1 seroconversion, defined as a fourfold or greater rise in titer between pre- and post-injection samples, at day 28 was observed in >96% of subjects in vaccine group, but in none of the subjects in the placebo group (Table 5). GMTs at day 28 were 3,309 in the 5.0 log_10_ PFU group, 2,331 in the 4.0 log_10_ PFU group and 1,367 in the 3.0 log_10_ PFU group, showing a trend for increased neutralizing antibody titers with increased doses of vaccine. Based on this immunogenicity and viremia findings the 5.0 log_10_ PFU formulation was selected for Part 2. 

In Part 2, seroconversion was achieved at day 28 by approximately 96% of subjects in both age cohorts in the vaccine group but none of the subjects in the placebo group. GMTs in 41–64 year-old subjects were 883 and 965 among subjects aged ≥65 years (Table 5). It appeared that, as was the case for the YF 17D vaccine [31], the antibody response is not diminished in the elderly. Titers dropped in all age groups at 12 months after vaccination; however, GMTs remained higher among the ≥65 years cohort (221) compared to the 41–64 years cohort (58). The persistence of antibodies after 12 months indicates that one dose of ChimeriVax-WN02 elicits a long-term antibody response, which in addition to the cellular immune response observed, may suggest that a one-dose vaccination schedule could be adequate for protection in the long-term. Long term immunity is expected as demonstrated in a randomized double-blind, 5-year phase II study in healthy adults with the similar ChimeriVax-JE (IMOJEV) [32]. 

#### 4.2.2. WN004 study

This was a randomized, double-blind, placebo-controlled, multi-center dose-ranging study in healthy adults conducted in eleven states in the midwest, west, and south of the US, most of them reporting WN virus activity [26]. The study was conducted among subjects ≥50 years of age who were healthy or had medically-stable pre-existing conditions. Subjects were vaccinated with ChimeriVax-WN02 4 × 10^3^ PFU (3.0 log_10_ PFU) (n = 122 [n = 72 50–64 years, n=50 ≥65 years]), ChimeriVax-WN02 4 × 10^4^ PFU (4.0 log_10_ PFU) (n = 124 [n = 75 50–64 years, n = 49 ≥ 65 years]), ChimeriVax-WN02 4 × 10^5^ PFU (5.0 log_10_ PFU) (n = 112 [n = 62 50–4 years, n = 50 ≥ 65 years]), or placebo (n = 120 [n = 76 50–64 years, n = 44 ≥ 65 years]). A subset from each group was randomized in a 2:1 ratio (viremia group : non-viremia group) to assess vaccine viremia. 

##### 4.2.2.1. Safety

This study confirmed the good safety and tolerability profile of ChimeriVax-WN02 in the vaccinated groups compared to placebo. In this study, subjects were followed during 6 months for safety. All groups reported similar frequencies of unsolicited AEs or ARs. The percentage of subjects reporting at least one unsolicited non-serious AE ranged between 38% (5.0 log_10_ PFU group) and 45% (4.0 log_10_ PFU group). Most unsolicited AEs were common infections such as nasopharyngitis, and were mild or moderate in severity. AR ranged between 9% (5.0 log_10_ PFU group) and 18% (4.0 log_10_ PFU group). Most unsolicited ARs were reported in the System Organ Class (SOC) of general disorders and administration site conditions, being fatigue the most commonly reported AR in this SOC. All SAEs and deaths were considered as not related to the study vaccine. The good safety profile among the elderly was also confirmed in this study where no statistically significant differences in terms of safety were found between both age groups (*i.e.*, 50–64 years and ≥65 years).

##### 4.2.2.2. Viremia

ChimeriVax-WN02 was associated with a low and transient viremia, similar to findings in prior studies after vaccination with this vaccine [24,26], YF 17D vaccine [10] and other recombinant, chimeric, live attenuated vaccines such as ChimeriVax-JE [28], or dengue [33,34,35,36]. In general, no differences in the proportion of subjects with vaccine viremia, measured by plaque assay with immunostain with a WN virus envelope protein specific monoclonal antibody, were observed among the vaccine groups in this study. Mean daily viremia levels were low and the peak viremia and mean AUC were similar across all 3 groups (Table 4). By contrast, an inverse relationship between viremia and dose was observed in prior studies with this vaccine [24,26], YF 17D vaccine [11] and ChimeriVax-JE vaccine [28]. This paradoxical response may be due to a lower innate and delayed adaptive immune response to the lower dose of vaccine and has been described with the YF 17D vaccine [37]. Viremia was detected between days 2 and 14 in active vaccine groups, tended to peak around days 4 and 6, and decreased to a frequency of <2% on Day 14. No subjects with viremia at day 14 had viremia at day 28. In general, the proportion of viremic subjects tended to be higher in the ≥65 year group. The mean duration of detectable viremia in subjects vaccinated with 5.0 log_10_ PFU aged ≥65 years (3.6 days) was longer than in subjects aged 50–64 years (2.8 days). However, the duration of quantifiable viremia appeared to be higher in those aged 50–64 years (5.8 days) vs. those aged >65 years (3.2 days). In addition, and different to the prior study, the peak viremia and mean AUC was not markedly higher in the ≥65 years subjects compared to subjects aged 50–64 years (Table 4). This finding is reassuring since elderly subjects are at higher risk of severe adverse events associated with YF 17D vaccine [38], reflecting a more active infection (higher level of *in vivo* virus replication) or senescent immune response in this age group.

##### 4.2.2.3. Immunogenicity

Similar to prior studies, high seroconversion rates were observed. Seroconversion, defined as a four-fold or greater rise in titer between pre- and 28 day post-injection samples, was achieved by ≥92% of the subjects in the active vaccine groups. The GMTs at day 28 ranged between 600 and 688, in the vaccine groups (Table 5) but did not increase in the placebo group (GMT 6). Although not statistically significant, slightly higher GMTs were observed in the older age group. These results seem to be promising particularly for the elderly who may benefit the most with WN vaccination since the risk of more severe disease is increased in this group. Neutralizing antibodies are well established as the mediator of protection against flavivirus infections, but the protective level of antibody has not been established for WN disease. In the case of JE, a similar disease caused by a closely related flavivirus, however, the seroprotective level of neutralizing antibody is 1:10 [39]. ChimeriVax-WN02 elicited antibody titers that are considerably above a PRNT titer of ≥10. 

## 5. Environmental Risk Assessment

A theoretical concern with any live replicating vaccine against a vector-borne disease is whether the vaccinated host could serve as the source of infection of blood-feeding arthropods in nature. This could provide an uncontrolled setting in which mutations could occur in the virus that alter virulence and facilitate transmission to vertebrate species. One safeguard is the low viremia observed in clinical studies, since in general oral infection of mosquitoes with flaviviruses requires a threshold viremia of approximately 3.0 log_10_ PFU/mL for infection of 1% of highly susceptible mosquito species and 5.0 log_10_ PFU/mL for efficient infection [40], which is substantially higher than seen for ChimeriVax-WN02. Moreover, it has long been known, that YF 17D virus, the backbone of the chimeric vector, is unable to be transmitted by mosquito vectors [41]. Similar results were observed in mosquitoes that were intrathoracically inoculated or had orally ingested the ChimeriVax-JE vaccine [42]. To assess the ability of the ChimeriVax-WN virus to replicate in mosquitoes, various species were inoculated by the intrathoracic route (which bypasses the midgut epithelial barrier) [43]. Infection was determined by testing the mosquito body for virus after a suitable extrinsic incubation period, and the potential for transmission was determined by testing head tissue (salivary glands) for virus. ChimeriVax-WN 01 (wild-type sequence veterinary vaccine, which replicates better than ChimeriVax-WN02 by various tests in vertebrate species) and YF 17D viruses did not replicate or disseminate to salivary glands in *Culex tritaeniorhynchus* or *Cx. nigripalpus*, and replication and dissemination were restricted in *Cx. quinquefasciatus*, *Ae. aegypti*, and *Ae. albopictus* compared to wild-type WN virus. To assess the ability of ChimeriVax-WN01 to be transmitted by mosquitoes, various species were fed blood meals containing virus and tested after a suitable extrinsic incubation period. None of the *Culex* mosquitoes, the primary vectors for WN virus, were infected orally with ChimeriVax-WN01 virus; one *Ae. albopictus* and 10% of the *Ae. aegypti* became infected, but the titer was very low and virus did not disseminate to head tissue. These results were similar to those with YF 17D virus and other ChimeriVax vaccines. In contrast, wild-type WN virus efficiently infected all mosquito species by the oral route [43]. The inefficient oral infection and dissemination to the salivary glands of mosquitoes represent the second barrier preventing uncontrolled spread of ChimeriVax-WN in nature. 

The virus also failed to infect chickens and fish crows. This indicated that avian species, the primary natural hosts of WN virus, will not be able to similarly harbor ChimeriVax-WN [44]. Altogether, the chimera is highly unlikely to enter a natural transmission cycle with mosquito vectors and birds as amplifying hosts. 

There have been several reports of shedding in urine of RNA genomes of YF 17D after yellow fever vaccination [45] and of wild-type WN after natural infection [46], although the findings of the latter study could not be replicated by others [47]. Shedding of genomes has been detected at long intervals after infection, suggesting that chronic infection occurs in humans [45]. Chronic infection with WN virus has also been repeatedly demonstrated in nonhuman primates and hamsters infected with WN [48], and the kidney appears to be a target organ. However, no live virus has ever as yet been recovered from urine of human patients, reflecting containment of the infection by the immune response. In addition, secondary spread of wild-type WN virus or of YF17D to contacts has never been reported. Therefore, the associated risk of spread of ChimeriVax-WN through urine appears to be negligible.

Further, the chances of reversion of ChimeriVax-WN virus to virulence are considered to be low because of a variety of factors discussed previously [18]. Numerous simultaneous mutations, *i.e.*, reversions to wt sequence in both the structural and NS proteins, would be required, which is unlikely. The YF 17D vaccine is known to be phenotypically stable as no new virulent YF virus strains have emerged as a result of its wide use in the course of over seven decades. The latter fact is in part due to a characteristic feature of the YF 17D RNA polymerase, also utilized in ChimeriVax vaccines, which is its high fidelity [49]. 

Finally, the likelihood of recombination of ChimeriVax-WN with other endemic flaviviruses resulting in new pathogens is considered remote [17,18]. In contrast to some other plus-strand RNA viruses, such as alphaviruses and picornaviruses, flaviviruses are not prone to recombination in nature or experimental settings [50,51]. In addition, several studies that examined “the worst-case scenario” of recombination between ChimeriVax vaccines and wild type JE, Kunjin, DEN4, SLE and YF Asibi viruses indicated that such recombinants, if they ever emerged, would have biological characteristics closer to the highly attenuated YF 17D parent than existing flavivirus pathogens and would be unlikely to successfully compete with endemic flaviviruses for survival in nature [52,53,54]. 

## 6. Challenges for Late Clinical Development and Licensure of a West Nile Vaccine

The WNV vaccine was developed in response to the emergence of WN fever and WN encephalitis as important endemic and epidemic diseases in North America from 1999 onwards. WN has caused substantial morbidity and mortality in repeated outbreaks since its introduction, and a vaccine represents a potentially important public health measure. Nevertheless, the relatively low incidence along with the sporadic and unpredictable nature of WNV activity, and the high inapparent:apparent infection ratio make the demonstration of field efficacy difficult. This results in a number of associated challenges for the clinical development of the vaccine as well as the licensure pathway. 

The epidemiology of the disease presents some difficulty in pursuing a classical licensure pathway, including a clinical efficacy trial. An alternative licensure strategy could be developed in collaboration and partnership with health authorities. Licensure considerations such as the classification of WN vaccine as an Orphan Drug or the pursuit of licensure under Animal Rule provisions are viable options for evaluation. For example, it might be possible to establish in addition to the safety and immunogenicity of the vaccine, a surrogate correlate of protection through the use of passive antibody transfer experiments in an animal model. The use of neutralizing antibodies as the appropriate surrogate will require the development of sufficient data to show that a specific antibody level is reasonably likely to predict clinical benefit. The laboratory standard assessment of the immune response in humans is the PRNT. This measure has been used successfully to estimate the efficacy of JE vaccines; however, recent data from a dengue efficacy trial [55] indicates that the PRNT as a measure of neutralizing antibody will need to be carefully evaluated for each flavivirus to determine its ability to estimate efficacy or to be used to define a surrogate marker of efficacy. In a recent study of the correlate of protection against YF virus, a higher level of antibody (1:40) was determined for complete protection [56], and a titer of 1:50 was estimated to be required for protection against dengue [57]. Of note, these titers have not yet been established as correlates of protection for dengue. However, it is reasonable to consider all of the available flavivirus protection data to estimate the serological level that may be required to provide protection. Since WN virus causes a systemic disease similar to dengue as well as neuroinvasion and encephalitis, it is possible that neutralizing antibody levels greater than 1:10 (established for JE) may be required. Further, it may be necessary to consider the evaluation of the impact between pre-existing flavivirus immunity (such as YF vaccination or exposure to SLE virus) on post WNV vaccination neutralizing antibody levels.

In addition to a surrogate marker of protection, the clinical development of the vaccine may require an expanded safety and immunogenicity clinical study to demonstrate sufficient neutralizing antibody levels (based on surrogate levels found protective in animal studies) and epidemiological data to demonstrate that the vaccine is reasonably likely to provide the clinical benefit of protection from serious WN associated fever and/or neurological disease. It may also be necessary to evaluate any potential interactions with other vaccines either pre- or post-licensure (e.g., influenza, tetanus-diphtheria, herpes zoster). In the absence of a clinical efficacy trial, the clinical benefit of vaccination may also need to be demonstrated in post-licensure effectiveness studies.

A considerable challenge for the licensure of the WN vaccine is the likelihood of acceptance of a clinical strategy for licensure that does not include a clinical efficacy trial. It has been postulated that Animal Rule provisions in the USA regulations could be a possible licensure strategy for WN vaccines. However, to date, no vaccine has been accepted to utilize this provision for licensure, and it seems the provisions have been developed principally to support development of countermeasures against bioterrorism. It is worth noting in the context of the animal rule, however, that the veterinary vaccines against WN were approved based on protection against experimental challenge of horses and immunological measurements, despite the incidence of WN in horses exceeding 650 per 100,000. Interestingly, two of the three approved vaccines (Table 2) elicit relatively weak neutralizing antibody responses (PRNT50 GMT 5-26) and yet are highly effective against a severe intrathecal challenge with virulent WN virus [13]. The pre-challenge antibody titers are significantly lower in the equine host than in humans following vaccination with ChimeriVax-WN02. 

A pragmatic and collaborative approach to the clinical development and licensure by vaccine developers and health authorities will be necessary for vaccines such as WN, whose unique epidemiology so significantly impacts the options for licensure. The licensure process of the WN vaccine could ultimately benefit from the FDA’s Advancing Regulatory Science. This initiative was launched in 2010 to develop new tools, standards, and approaches to assess the safety, efficacy, and quality of novel medical products due to the rapid advances in innovative science in the last few years [58]. In addition, strategies developed for WN virus could also be applicable to future emerging infectious diseases. Vaccination recommendations would also need careful consideration. Weak or permissive vaccination recommendations would likely result in poor uptake. Under these circumstances, it is difficult for any manufacturer to justify an extensive development program or to build production capacity, even if there were a clear licensure pathway. A cost-benefit analysis to determine what groups would benefit from the vaccine could be very useful to support the development of a WN vaccine and subsequently strengthen vaccination recommendations.

## 7. Conclusions

The available data on the safety and immunogenicity of WN02 candidate vaccine can be considered supportive for vaccine licensure. The pre-clinical studies have demonstrated that the WN vaccine is less neurovirulent than YF 17D, elicits neutralizing antibodies in various animal models, and protects immunized animals from challenge with high doses of wild type virus. Three human clinical trials have confirmed the good safety profile of WN vaccine as compared to placebo, with similar frequencies of unsolicited AEs or ARs, and most AEs being categorized as mild or moderate in severity. In the elderly population, a similar good safety profile was established as compared to adults. The vaccine elicited a strong humoral and cellular immunogenicity, and vaccination with WN vaccine was associated only with a low and transient viremia. In addition, environmental risk assessment evaluation of possible issues of reversion to virulence, recombination or transmission by arthropod vectors has not indicated significant concerns. The epidemiology of the disease presents some difficulty to pursue a classical licensure pathway. The challenges for the development of this vaccine highlight the importance of exploring “non-classical” licensure pathways for a new emerging/reemerging disease without a standard Phase III efficacy trial. The licensure process of the WN vaccine could ultimately benefit from the recent innovative FDA’s Strategic Plan for Regulatory Science. Assessment of different licensure/vaccination options in parallel to the epidemiology and disease burden will certainly require a close collaboration with the involved health authorities to make a human WN vaccine a reality.
